# Impact of anakinra use on clinical outcomes in children with moderate or severe multisystem inflammatory syndrome in children: a propensity score matched retrospective cohort study

**DOI:** 10.1186/s12969-023-00924-6

**Published:** 2023-11-23

**Authors:** Esra B. Akkoyun, Zachary Most, Harita Katragadda, Andrew Yu, Lorien Nassi, Nicole Oakman, Sarah Ginsburg, Mia Maamari

**Affiliations:** 1 Department of Pediatrics, Children’s Medical Center, 1935 Medical District Dr. Dallas, Dallas, TX USA; 2 University of Texas Southwestern, Dallas, TX USA

**Keywords:** MIS-C, COVID-19, Children, Anakinra

## Abstract

**Background:**

The treatment of children with multisystem inflammatory syndrome in children (MIS-C) related to SARS-CoV-2 infection involves immunomodulatory therapies such as IVIG and steroids. Anakinra, an interleukin-1 receptor inhibitor, has also been used, but its effectiveness is not established yet. As optimal regimens for MIS-C remain unknown, we aimed to assess the effect of anakinra in reducing hospital stay in patients with MIS-C.

**Methods:**

We included children admitted from May 2020 to May 2021 diagnosed with MIS-C based on CDC criteria. The exposure of interest was anakinra use at any point during admission. The anakinra exposed group and the anakinra unexposed group were propensity score matched based on demographic and clinical severity indicators at initial presentation. Our primary outcome was length of hospital stay. Secondary outcomes were duration of vasoactive support, vasoactive inotropic score (VIS), level of respiratory support, time to fever resolution, reduction of CRP levels, and length of ICU stay. We used Wilcoxon rank sum, t-test, Chi square and Fisher’s exact tests.

**Results:**

Of 138 children diagnosed with MIS-C, 79% had moderate or severe illness and 41% received anakinra. Of those, 31 patients who received anakinra were propensity score matched to 31 who did not. The length of stay in the hospital but not in the ICU was longer in the anakinra group. There were no differences in median duration of vasoactive support, fever resolution, CRP reduction, or VIS.

**Conclusions:**

In patients with moderate to severe MIS-C, use of anakinra was associated with longer duration of hospital stay.

## Background

In April 2020, multisystem inflammatory syndrome in children (MIS-C) emerged as a rare but serious condition temporally associated with SARS-CoV-2 infections [1–8]. Signs and symptoms include fever, malaise, abdominal pain, fatigue, poor appetite, rash, and conjunctivitis, with involvement of two or more organ systems, and typically develop 4–6 weeks after acute infection with SARS-CoV-2 [1, 9]. Patients with MIS-C demonstrate a hyperinflammatory laboratory profile [3, 5, 6, 8, 10] with cardiovascular involvement, including shock, echocardiographic findings of decreased ventricular function, and less frequently coronary artery aneurysms [11]. Given the overlap in clinical and lab findings with Kawasaki disease (KD), therapies for MIS-C were adapted from KD management, including the use of intravenous immunoglobulin (IVIG) and other anti-inflammatory or immunomodulatory agents such as steroids [3, 12, 13].

In cases of resistant KD, shock syndrome, worsening coronary artery dilation, or life-threatening myocarditis, anakinra can be added to inhibit interleukin-1 (IL-1) [13, 14] since IL-1 is involved in the pathogenesis of coronary artery aneurysms [15]. Clinical and experimental evidence suggests a key role for IL-1α in myocardial inflammation and contractile dysfunction following myocardial injury [14, 16]. A phase II open-label study demonstrated safe use of anakinra in resistant KD and its effect on reducing fever, inflammatory markers, and coronary artery dilation [17].

Children with MIS-C have elevated levels of systemic inflammatory markers and can rapidly decompensate with multiorgan dysfunction, including hypotension and cardiac dysfunction [10, 18]. Similar to high-risk KD, some patients with moderate or severe MIS-C may benefit from additional immunomodulatory therapies beyond IVIG and/or steroids. Many centers have adopted a combination of IVIG, steroids, and immunomodulators such as infliximab (tumor-necrosis-factor inhibitor), tocilizumab (interleukin-6 inhibitor), or anakinra [9, 19, 20].

There is considerable variability in the management of MIS-C and no evidence-based consensus to indicate the most effective treatment strategy. Therefore, we hypothesized that treatment with anakinra in addition to IVIG and steroids may improve outcomes in those with moderate or severe MIS-C.

## Methods

### Study population

This was a retrospective, single-institution observational cohort study conducted at a quaternary care pediatric healthcare system from May 2020 to May 2021. Individuals were included if they were under 21 years old, admitted to the hospital and were diagnosed with MIS-C by 2020 Centers for Disease Control and Prevention criteria [21]. Cases were identified from three sources: review of all admitted patients during the study period for MIS-C diagnosis codes, an infectious disease consult running list of MIS-C patients, and critical care provider review of all patients admitted to the intensive care unit.

We extracted data from the electronic health record via systematic retrospective chart review, which included socio-demographic information, clinical characteristics, laboratory and cardiac evaluations, medications, and outcomes as defined below. Data collection and management were conducted by using REDCap (Vanderbilt University, Nashville, TN) [22]. The study was approved by the University of Texas Southwestern Medical Center Institutional Review Board with a waiver of informed consent.

### Classification and treatment of MIS-C

We classified MIS-C severity depending on the level of respiratory or hemodynamic organ support and end organ injury (Table 1). Patients with no or minimal respiratory or cardiovascular support and minimal end organ injury were categorized as mild MIS-C. Those with no or minimal respiratory or cardiovascular support and mild or isolated end-organ injury were categorized as moderate MIS-C. Those requiring any respiratory support, vasoactive or inotropic support, or with severe or multi-organ injury were categorized as severe MIS-C.

**Table 1:** Classification of MISC and Methylprednisolone Dosing Based on Severity

	Mild	Moderate	Severe (ICU care)
Respiratory support	None	None or minimal or significant	None or minimal or significant
Cardiovascular support	None	None or minimal	Vaso actives or inotropes
End organ injury	Minimal	Mild or isolated	Severe or multi-organ
Initial dose (mg/kg/day) Maintenance daily dose	None or minimal	10 mg/kg (max 1 g) x 1–3 days 2 mg/kg (max 60 mg) x 5 days	20-30 mg/kg (max 1 g) x 1–3 days 2 mg/kg (max 60 mg) x 5 days

The management of MIS-C was based on the severity of illness. Therapy typically incorporated IVIG and varying doses of methylprednisolone depending on disease severity. Those with mild disease received 2 mg/kg/day methylprednisolone for 3–5 days, those with moderate disease received 10 mg/kg/day for 1–3 days, while those with severe disease received 20–30 mg/kg/day for 1–3 days (Table 1). In the moderate and severe groups, this was followed by an oral steroid tapering regimen.

Our institutional protocols for MIS-C changed over the study period. Over time, anakinra was used earlier in the disease course in severe patients and some moderate patients. The decision to initiate anakinra, along with its dosing and duration, was based on institutional guidelines and consultation with a pediatric rheumatology specialist. Anakinra was administered subcutaneously or intravenously, with dosing tailored to the severity of the disease, ranging between 2 and 10 mg/kg/day or 100-400 mg/day with a maximum of 400 mg/day. Anakinra was typically given between the first and second echo. Treatment was subsequently tapered off by the time of discharge.

Anakinra was used either as a primary or additional therapy during hospitalization. Initial therapy was defined as administration of IVIG or first dose of steroids. Additional therapy was defined as administration of an immunomodulatory agent more than 24 h after treatment initiation of either IVIG and/or steroids (first dose). Additional therapies were prescribed for any of the following indications: fever (38.0 °C or greater) occurring more than 24 h after finishing initial therapy, a new onset fever after 24 h period of initial improvement following the administration of intravenous immunoglobulin (IVIG) or steroids, continued need for vasoactive medications, worsening echocardiogram findings, and/or laboratory evidence of persistently high or worsening markers of inflammation including CRP, D-dimer, and ferritin levels.

Time to immunomodulatory agent was described as the duration in hours from arrival to our institution to initiation of the immunomodulatory agent including IVIG, steroids and anakinra. Rebound fever was defined as new onset fever > 24 h after IVIG was initiated.

### Outcomes

The primary outcome was length of hospital stay measured in hours between the time and date of presentation to our health system (either to the emergency department or inpatient wards or intensive care unit) and the time and date of discharge, which is when the patient physically left the room. Secondary outcomes included the duration of vasoactive support (including epinephrine, vasopressin, norepinephrine and milrinone) measured as the time between the initiation of any vasoactive agent and the discontinuation of all vasoactive agents, the maximum daily vasoactive-inotropic score (VIS), the highest level and duration of respiratory support (number of calendar days), the duration of fever (number of calendar days), C-reactive protein (CRP) reduction, length of ICU stay, and new or worsened left ventricular (LV) dysfunction or coronary artery aneurysms. VIS is a clinical tool used in critically ill patients to quantitatively assess their cardiovascular support requirements. It involves adding up the potency-weighted doses of vasoactive and inotropic agents. Reduction of CRP was defined as the percentage decrease of daily CRP levels. New or worsened LV dysfunction was defined as newly developed left ventricular shortening function (LVSF) < 30% at least 24 h after treatment initiation, or worsening LVSF at least 24 h after treatment initiation. Coronary artery dilation or aneurysm was defined as new if it was present on repeat echocardiogram at least 24 h after treatment initiation and was not present on initial echocardiogram. Rebound fever, described as the fever returns after 24-hour period of initial improvement following the administration of intravenous immunoglobulin (IVIG) or steroids.

### Propensity score generation and statistical analysis

Demographic and diagnostic variables at time of admission that had some association (*p* ≤ 0.10) with the administration of anakinra were incorporated into a multiple logistic regression model to create a propensity score. The following baseline variables were obtained before initial MIS-C therapy and were used in the propensity score: presence of left ventricular dysfunction measured as LVSF < 30% on initial echocardiogram, quantitative measurement of LVSF, severity of MIS-C and level of high sensitivity troponin. The “greedy nearest-neighbor” matching algorithm without replacement was used to match observations based on their propensity score. This algorithm began pairing observations in descending order of their propensity score and employed a 1:1 ratio within a caliper width of 0.5 of the standard deviation of the logit of the propensity score.

Patients who received anakinra were matched by their propensity score to those who did not receive anakinra. These two groups were subsequently compared. Continuous variables were expressed as medians and interquartile ranges, while categorical variables were expressed as frequencies and proportions. Normality was assessed using the Shapiro-Wilk test. The Wilcoxon Rank Sum test was employed for non-normal distributions, and a two-sample t-test was used for normal distributions. Categorical variables were examined for associations by the Chi-square test or Fisher’s exact test. Statistical analysis and propensity-score matching were performed with SAS 9.4 (SAS Institute Inc, Cary, NC) and R studio version 1.4.1106 (RStudio Team (2020) Boston, MA).

## Results

### Demographic and clinical characteristics of all patients

**Fig. 1:**
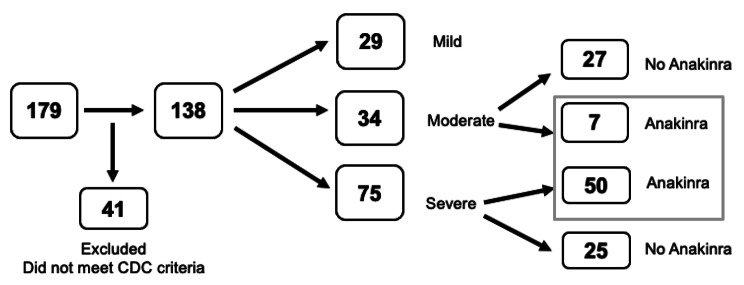
Study flowchart

Among 179 pediatric patients with suspected MIS-C, 138 fulfilled 2020 CDC criteria for MIS-C associated with SARS-CoV-2 infection (Fig. 1).

While 54% received respiratory support, 84% of those received low level support such as nasal cannula, venturi mask or heated high flow nasal canula. Only 8 patients (6%) required intubation and the median duration of intubation was 5.2 days. Almost half (49%) of patients required vasoactive support, with the median duration of vasoactive support of 20.6 h. A total of 94% of patients received IVIG, with 7% receiving a second dose of IVIG. Nearly all patients (93%) received steroids and 57 patients (41%) received anakinra. Thirty-four patients (25%) demonstrated LV dysfunction, with a median LV shortening fraction of 32% (28–36%). Twelve patients (9%) had coronary artery aneurysm at presentation. Ninety patients (65%) required ICU care, with a median length of stay in the ICU of 2.0 days, and in the hospital of 6.3 days (Table 2).

**Table 2:** Demographic and clinical characteristics of all patients

Characteristics	All (*N* = 138)
Age (years), median (IQR)	9.6 (7.2–13.0)
Female sex, *n* (%)	63 (46)
Weight (kg), median (IQR)	41.5 (26.6–61.7)
**Race/Ethnicity,** *n* (%)	
Asian	4 (3)
White non-Hispanic	33 (24)
Hispanic	50 (36)
Black non-Hispanic	49 (36)
Other	2 (1)
**Comorbidities,** *n* (%)	
Any co-morbidities	60 (43)
Asthma	19 (32)
Obesity	18 (30)
Other (anemia, hydronephrosis, ADHD, autism)	38 (63)
**Severity of MIS-C,** *n* (%)	
Moderate	34 (25)
Severe	75 (54)
**Organ support**	
Respiratory support, *n* (%)	74 (54)
Nasal cannula or venturi mask	44 (59)
High flow nasal cannula	18 (24)
Continuous or bi-level positive airway pressure	4 (5)
Intubation	8 (11)
Duration of intubation (days), median (IQR)	5.2 (4.1-6.0)
Duration of all respiratory support (days), median (IQR)	3 (1–6)
Vasoactive support, *n* (%)	69 (50)
Duration of vasoactive use (hours), median (IQR)	20.6 (10.4–48.3)
**Echo findings**	
Presence of LV dysfunction, *n* (%)	34 (25)
LV shortening fraction (%), median (IQR)	32 (28–36)
Coronary artery aneurysm, *n* (%)	12 (9)
**Immunomodulator therapy**	
IVIG, *n* (%)	129 (93)
Second dose of IVIG, *n* (%)	10 (7)
Methylprednisolone, *n* (%)	136 (99)
Anakinra, *n* (%)	57 (41)
Duration of anakinra (days), median (IQR)	5.5 (4.0-6.2)
**Outcomes**	
Fever duration during admission (days), median (IQR)	1.0 (1.0–2.0)
ICU stay, *n* (%)	90 (65)
Length of ICU stay (days), median (IQR)	2.0 (1.4–4.9)
Length of hospital stay (days), median (IQR)	6.3 (4.2–8.7)

ADHD: attention deficit hyperactivity disorder; ICU: intensive care unit; IQR: interquartile range

IVIG: intravenous immunoglobulin; LV: Left ventricle

### Baseline characteristics of matched cohort

**Fig. 2:**
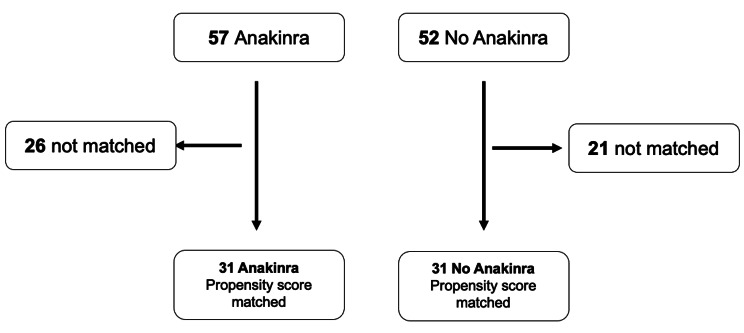
Propensity Score Matching of Children with Multisystem Inflammatory Syndrome in Children (MIS-C)

Figure 2 shows numbers of matched and unmatched patients.

Table 3 compares baseline characteristics and demographics for the anakinra vs. no anakinra groups, both before and after propensity score matching. In the propensity score matched cohort, severe MIS-C was diagnosed in 77% of both groups.

**Table 3:** Demographic and clinical characteristics of anakinra vs. no anakinra groups before and after propensity score matching

	Before propensity score matching			After propensity score matching		
Demographics	Anakinra (*N* = 57)	No Anakinra (*N* = 52)	*p* value	Anakinra (*N* = 31)	No Anakinra (*N* = 31)	*p* value
Age (years), median (IQR)	9.9 (8.1–13.9)	9.4 (7.2–11.7)	0.13	8.8 (5.8–12.9)	9.5 (7.8–11.4)	0.89
Female, *n* (%)	25 (44)	24 (46)	0.8	14 (45)	15 (48)	0.8
**Race/Ethnicity,** *n* (%)			0.67			0.26
Asian	1 (2)	2 (4)		0 (0)	1 (3)	
White non-Hispanic	8 (14)	12 (23)		7 (23)	10 (32)	
Hispanic	23 (40)	20 (38)		16 (52)	9 (29)	
Black non-Hispanic	22 (39)	15 (29)		8 (26)	10 (32)	
**Comorbidities,** *n* (%)	28 (49)	19 (37)	0.18	15 (48)	11 (35)	0.3
Asthma	9 (16)	8 (15)	1	3 (10)	5 (16)	0.71
Obesity	9 (16)	5 (10)	0.39	3 (10)	3 (10)	1
**Clinical Characteristics (at presentation)**						
**Temperature (** ^**o**^ **C)**						
Temperature, median (IQR)	38.9 (38.2–39.6)	39.1 (38.3–39.5)	0.2	39.0 (38.3–39.7)	38.9 (38.2–39.5)	0.98
Presence of fever, *n* (%)	56 (98)	50 (96)	0.73	25 (80)	26 (84)	0.73
**Laboratory markers, median (IQR)**						
CRP (mg/dL)	18.6 (14-22.5)	14.2 (9.6–21.4)	0.07	16.4 (12.8–21.7)	19.0 (12.7–24.1)	0.43
High sensitivity troponin (pg/mL)	129.5 (14.7–273)	27.4 (2.5–97.1)	**0.002**	14.7 (3.1–60.9)	27.4 (2.5–99.0)	0.98
Creatinine (mg/dL)	0.6 (0.4-1.0)	0.5 (0.4–0.8)	0.35	0.57 (0.42–0.74)	0.60 (0.47–0.91)	0.66
ALT (units/L)	43 (19–65)	29 (18–65)	0.43	45 (20–73)	30 (20–75)	0.74
D-dimer (mcg/mL FEU)	3.4 (2.3–6.1)	2.7 (1.5–4.2)	0.06	3.2 (2.4–5.6)	2.7 (2.0-4.9)	0.32
Ferritin (ng/ml)	482 (280–1166)	333 (191–978)	0.05	525 (354–1193)	584 (239–1026)	0.69
**Severe classification,** *n* (%)	49 (86)	26 (50)	**0.00005**	24 (77)	24 (77)	1
**Immunomodulatory treatment, median (IQR)**						
Time to IVIG (hours)	12.1 (8.9–22.5)	18.4 (10.9–30.6)	**0.03**	14.1 (9.9–32.4)	17.6 (9.7–23.2)	0.94
Time to steroids (hours)	11.5 (6.5–20.5)	18.5 (8.1–32.7)	**0.03**	12.9 (7.7–25.3)	15.8 (7.8–28.7)	0.69
Time to anakinra (hours)	23.8 (14.6–37.9)	NA	NA	26.1 (14.6–56.8)	NA	NA
Anakinra duration (days) Time from last anakinra	5.5 (3.3–5.8)	NA	NA	4.9 (3.8–7.2)	NA	NA
dose to discharge (hours)	47.4 (28.0–59.0)	NA	NA	47.4 (25.9–58.7)	NA	NA

There was no statistically significant difference between the matched groups relative to baseline variables not used in the propensity score. There was also no difference in age, gender, or co-existing conditions between the two matched groups. The median temperature on admission was 39.0 °C in the anakinra group, 38.9 °C in the no anakinra group. Moreover, no differences were observed in the initial laboratory tests including CRP, D-dimer, ALT, ferritin, and creatinine between the two matched groups.

Regarding echocardiogram findings at hospital admission (Table 3), 29% of both cohorts had left ventricular dysfunction. The median LVSF was the same in both groups, at 32%. Five patients (16%) in the anakinra group had coronary artery dilation, which was not different when compared to zero of those without anakinra.

In the propensity score matched groups, the median time from presentation to first IVIG administration was 14.1 h in the anakinra group vs. 17.6 h in the no anakinra group with no difference. The median time to steroids was 12.9 h in the anakinra group vs. 15.8 h in the no anakinra group with no difference. The median duration of steroid use was 5 days in the anakinra group vs. 4 days in the no anakinra group with no difference. The median time from presentation to first anakinra administration in the anakinra group was 26.1 h. The median duration of anakinra use was 5 days.

### Outcomes of matched cohort

Table 4 compares outcomes in anakinra vs. no anakinra groups. The median length of hospital stay was longer in the anakinra group at 8.7 days compared to 6.1 days in the no anakinra group (*p* = 0.002). The median length of ICU stay was 1.8 vs. 2.1 days in the anakinra vs. no anakinra groups (*p* = 0.18).

**Table 4:** Outcomes of anakinra vs. no anakinra groups before and after propensity score matching

	Before propensity score matching			After propensity score matching		
Outcomes	Anakinra (*N* = 57)	No Anakinra (*N* = 52)	*p* value	Anakinra (*N* = 31)	No Anakinra (*N* = 31)	*p*-value
**Duration of organ support, median (IQR)**						
Vasoactive support (hours)	19.6 (8.9–55.7)	20.6 (12.5–31.3)	0.25	16.2 (8.3–48.5)	19.1 (11.7–34.2)	0.64
Respiratory support (days)	3.0 (2.0-5.5)	2.0 (1.0-6.8)	0.23	5 (1–8)	2 (1–6)	0.14
**Organ support,** *n* (%)						
Respiratory support	39 (68)	34 (65)	0.73	18 (58)	24 (77)	0.1
Vasoactive support	44 (77)	24 (46)	**0.0008**	21 (68)	22 (71)	0.78
VIS, day 1, median (IQR)	10.0 (6.0–15.0)	7.0 (5.0-10.2)	0.3	7.0 (6.0–13.0)	6.0 (5.0-9.5)	0.76
**Presence of fever**						
Fever duration during admission (days), median (IQR)	1 (1–2)	2 (1–3)	0.09	2 (1–3)	1 (1–3)	0.43
Max temperature (^o^C), day 2, median (IQR)	37.3 (36.7–38.3)	38.2 (36.9–39.1)	**0.01**	37.9 (37.2–39.1)	38.2 (36.9–38.9)	0.84
Fever, day 2, *n* (%)	19 (33)	27 (52)	**0.04**	15 (48)	18 (58)	0.44
Max temperature (^o^C), day 3, median (IQR)	37.1 (36.8–37.9)	37.2 (36.8–38.1)	0.23	37.3 (36.7–38.7)	37.2 (36.7–37.8)	0.5
Fever, day 3, *n* (%)	12 (21)	12 (23)	0.79	10 (32)	6 (19)	0.24
Fever, day 4, *n* (%)	6 (11)	13 (25)	**0.046**	5 (16)	8 (26)	0.34
Fever, day 5, *n* (%)	3 (5)	10 (19)	**0.024**	5 (16)	4 (13)	0.71
**CRP, median (IQR)**						
CRP, day 2	16.5 (13.9–21.0)	15.2 (10.9–19.9)	0.21	15.7 (13.3–19.9)	16.4 (11.8–20.3)	0.72
CRP, day 3	11.6 (7.8–17.0)	10.9 (7.9–16.8)	0.25	11.6 (8.7–18.6)	11.3 (8.4–16.9)	0.62
**VIS, median (IQR)**						
VIS, day 2	5.0 (4.3-8.0)	8.5 (4.0–16.0)	0.33	7.0 (6.0–13.0)	6.0 (5.0-9.5)	0.76
VIS, day 3	4.5 (2.0–7.0)	8.0 (5.8–10.0)	0.33	5.0 (4.0–8.0)	5.0 (2.0–11.0)	0.82
**Echo findings**						
LV dysfunction, *n* (%)	24 (42)	8 (15)	0.94	9 (29)	9 (29)	1
LVSF, median (IQR)	30 (24–33)	32 (29–35)	**0.01**	32 (28–35)	32 (26–34)	0.28
CAA, *n* (%)	5 (9)	2 (4)	0.29	5 (16)	0 (0)	0.05
**Length of stay (days), median (IQR)**						
ICU LOS	2.5 (1.3–4.3)	2.0 (1.6–5.9)	0.54	1.8 (1.1–5.5)	2.11 (1.7–6.2)	0.18
Hospital LOS	7.8 (6.7–10.2)	5.9 (4.4–8.4)	**0.00007**	8.7 (6.6–11.0)	6.1 (4.4–9.6)	**0.002**
Time from initial steroid dose to discharge	6.8 (6.0-9.6)	5.0 (4.0-6.8)	**0.00001**	8.1 (6.1–9.9)	5.2 (4.25–7.5)	**0.0008**

Vasoactive support was applied to 21 patients (68%) in the anakinra group and 22 patients (71%) in the no anakinra group. There were no differences in the proportion of matched subjects receiving organ support between the two groups. The median duration of vasoactive support was 16.2 vs. 19.1 h in anakinra vs. no anakinra group, which was not different. The median VIS on day 1 was 7 vs. 6 in the anakinra vs. no anakinra groups, respectively, with no statistically significant difference. The VIS score was not different between the two groups on day 2 and on day 3.

Respiratory support was applied to 18 patients (58%) in the anakinra group and 24 patients (77%) in the no anakinra group. The median duration of respiratory support was 5 vs. 2 days in anakinra vs. no anakinra group with no difference.

The median duration of fever following initiation of therapy (usually IVIG with or without steroids) was 2 days in the anakinra group, and 3 days in the no anakinra group (*p* = 0.43). 26% of patients in the anakinra group experienced rebound fever compared to 16% of those in the no anakinra group (*p* = 0.35). On hospital day 2, 48% of individuals in the anakinra group continued to have fever, while 58% of patients in the no anakinra group had the same (*p* = 0.44). The CRP reduction on hospital days 2 and 3 were not statistically significant between the two groups.

10% of those in the anakinra group developed new or worsened LV dysfunction which was likely an indication to use anakinra as a second line. Whereas only 3% of patients in the no anakinra group had this finding (*p* = 0.61). However, nearly all LV dysfunction resolved by hospital discharge.

## Discussion

In this retrospective cohort study of children hospitalized with moderate to severe MIS-C, hospital stay was longer in patients who received anakinra compared to patients who did not receive anakinra. Also, there was no difference in vasoactive support duration, VIS, fever duration, CRP reduction, or ICU length of stay in patients who received anakinra compared to patients who did not receive anakinra. This finding does not support improved outcomes with anakinra in the propensity score matched cohort.

### IVIG and steroids

The combination therapy of IVIG and glucocorticoids, when compared to IVIG alone, has been associated with a range of improved outcomes in patients with MIS-C. These include a more favorable fever course [23], lower frequency of treatment escalation [24], reduced need for hemodynamic support [24], shorter stays in the intensive care unit [9], a shorter time to recovery of cardiac function, and a lower risk of serious short-term outcomes [25]. A recent study showed evidence that administering glucocorticoids earlier in the disease process of MIS-C may limit the inflammatory cascade that leads to tissue injury, organ dysfunction, and shock, thereby leading to a faster recovery [26]. However, McArdle et al. did not find any evidence of differences in outcomes between treatment with glucocorticoids or IVIG as single agents, or between the single-agent and dual-agent primary treatments [27]. While there are discrepant results, the general recommendations are to include IVIG and steroids in the initial treatment of MIS-C.

### Non-anakinra biologics

Cole et al. found that patients with MIS-C who were treated with infliximab in addition to IVIG were less likely to receive supplementary treatment, had a shorter duration of ICU length of stay, had decreased development of LV dysfunction, and demonstrated a more rapid decline in CRP levels [19] than those who were only given IVIG. Notably in this study, steroids were not used as part of the treatment. Comparatively, in our study almost every child received steroids whether they also received anakinra or not. Our findings showed no difference in CRP reduction, worsening LV function, or ICU length of stay between the two groups. These findings highlight the potential benefits of combining different therapies in certain cases, and further emphasize the importance of individualized treatment plans based on patient needs and characteristics.

### Anakinra

Limited data exist regarding the use of anakinra in MIS-C, however a few case reports demonstrated clinical improvement, and a reduction in inflammatory markers [28, 29] as well as rapid cardiac function improvement [25] after anakinra initiation. A recent study showed that early anakinra treatment improves cardiac outcome regardless of disease severity [30]. Our findings showed no difference in CRP reduction or improving LV function between the two groups. Interestingly, our cohort had a higher proportion of patients (50%) with vasoactive need, whereas 19% in the Taddio study had hypotension or cardiogenic shock. Furthermore, their anakinra cohort (9%) was smaller than ours (41%). Given the different definitions of MIS-C by the Royal College of Paediatrics and Child Health [31] and the CDC [21], and the unstandardized definitions of severity, it is possible that the heterogeneous populations account for our different findings.

In our matched cohort, the anakinra group had a longer median length of hospital stay. Additionally, anakinra was not associated with improvement in vasoactive medication duration, time to fever resolution, organ support and ICU length of stay. The absence of statistically or clinically meaningful improved outcomes in the matched cohort suggests that anakinra, when given in addition to IVIG and steroids, does not improve patient outcomes in MIS-C. Since 93% of all patients received IVIG and 99% received steroids, the possibility of a confounding effect from those immunomodulators is minimized. However, with its relatively low risk profile, anakinra may still be beneficial in managing severe disease states.

To address the variability in factors that can impact time of admission, we performed a sensitivity analysis using length of stay with the starting from time of initial steroid administration to time of discharge and confirmed a longer stay in the anakinra group. The longer hospital stay in the anakinra group may be attributed to the duration of the taper and need for observation to monitor rebound signs and symptoms. Anakinra was administered with a delay of approximately 12–14 h following the start of IVIG or steroids and about 24 h after admission. This timing may be due to several factors: (1) anakinra may have been started in patients who exhibited an inadequate response to initial first-line therapies, (2) anakinra required a rheumatology specialist to order it, which may not have happened overnight with as much ease as ordering IVIG and steroids, and (3) clinician behavior changing over time, with later administration in disease course early in the pandemic but earlier adoption of anakinra later in the pandemic. This timing gap of 12–14 h may provide an additional explanation for the prolonged hospitalization seen in the anakinra group, but it would not account for the difference of 2–3 days. Interestingly, we found that there was about 48 h between the last dose of anakinra and time of discharge. This result in combination with the fact that ICU LOS was the same in the matched groups, suggests that the difference in hospital length of stay stems from the end of the hospital stay. This longer length of stay may be for clinical observation purposes once anakinra was stopped.

The main strength of this study is being one of the largest single institutional cohorts evaluating the effectiveness of anakinra use in MIS-C. In addition, the application of propensity score-matched analysis was important in controlling for confounding factors.

This study has several limitations. First, as a single center study in the USA from 2020 to 2021, the results may not be generalizable to other regions or countries, or to MIS-C in the future. Furthermore, the knowledge and familiarity with this new illness changed over time and it is impossible to ascertain how this may have affected practice patterns or patient outcomes. Second, it is unknown how the unmatched patients in the cohort may have impacted the results. It is possible that in severe but unmatched cases, anakinra was beneficial. Third, the dosage and duration of steroid and timing of anakinra treatment used was variable, and the small size of the study population precludes comparison of anakinra as first line or second-line treatment or by dose of anakinra. It is unclear if early use of anakinra as opposed to second-line use would lead to different outcomes. While our propensity score method can mitigate disparities between the groups at the study’s outset, they may not fully address discrepancies that may arise after admission. This scenario could introduce an unmeasured ‘confounding by indication’ bias that persists even after propensity score matching. Our primary outcome, length of stay was measured from time to arrival to our health system to time of discharge. While there may be unmeasured factors that affect time of discharge, such as social or logistical factors, they would likely similarly impact both anakinra and non-anakinra cohorts. Finally, severity classification has no quantitative thresholds and is based on expert consensus, which makes comparison of various studies difficult.

## Conclusion

Among children with moderate to severe MIS-C, anakinra use did not improve patient outcomes, but led to longer hospital stay compared to those who did not receive anakinra. Further investigation is necessary to evaluate and compare the effectiveness of various therapies for MIS-C.

## Data Availability

The datasets used and/or analyzed during the current study are available from the corresponding author on reasonable request.

## Abbreviations

ALT: Alanine aminotransferase
BMI: Body mass index
CDC: Centers for Disease Control and Prevention
CRP: C-reactive protein
CXR: Chest x-ray
GI: Gastrointestinal
HFNC: High flow nasal cannula
HsTroponin: High-sensitivity troponin
ICU: Intensive care unit
IQR: Interquartile range
IVIG: Intravenous immunoglobulin
KD: Kawasaki Disease
LOS: Length of stay
LRTI: Lower respiratory tract infection
LV: Left ventricle
LVSF: Left ventricular shortening fraction
MIS-C: Multisystem inflammatory syndrome in children
NC: Nasal cannula
VIS: Vasoactive inotropic score
VM: Venturi Mask
WBC: White blood cell
